# High-dose chemoradiotherapy followed by surgery versus surgery alone in esophageal cancer: a retrospective cohort study

**DOI:** 10.1186/1477-7819-8-46

**Published:** 2010-06-01

**Authors:** Meysan Hurmuzlu, Kjell Øvrebø, Odd R Monge, Rune Smaaland, Tore Wentzel-Larsen, Asgaut Viste

**Affiliations:** 1 Department of Oncology, Førde Central Hospital, N-6800 Førde, Norway; 2 Department of Surgery, Haukeland University Hospital, N-5021 Bergen, Norway; 3 Centre for Clinical Research, Haukeland University Hospital, N-5021 Bergen, Norway; 4 Department of Surgical Sciences, University of Bergen, N-5021 Bergen, Norway; 5 Department of Oncology and Medical Physics, Haukeland University Hospital, N-5201 Bergen, Norway

## Abstract

**Background:**

We aimed to assess whether high-dose preoperative chemoradiotherapy (CRT) improves outcome in esophageal cancer patients compared to surgery alone and to define possible prognostic factors for overall survival.

**Methods:**

Hundred-and-seven patients with disease stage IIA - III were treated with either surgery alone (n = 45) or high-dose preoperative CRT (n = 62). The data were collected retrospectively. Sixty-seven patients had adenocarcinomas, 39 squamous cell carcinomas and one undifferentiated carcinoma. CRT was given as three intensive chemotherapy courses by cisplatin 100 mg/m^2 ^on day 1 and 5-fluorouracil 1000 mg/m^2^/day, from day 1 through day 5 as continuous infusion. One course was given every 21 days. The last two courses were given concurrent with high-dose radiotherapy, 2 Gy/fraction and a median dose of 66 Gy. Kaplan-Meier survival analysis with log rank test was used to obtain survival data and Cox Regression multivariate analysis was used to define prognostic factors for overall survival.

**Results:**

Toxicity grade 3 of CRT occurred in 30 (48.4%) patients and grade 4 in 24 (38.7%) patients of 62 patients. One patient died of neutropenic infection (grade 5). Fifty percent (31 patients) in the CRT group did undergo the planned surgery. Postoperative mortality rate was 9% and 10% in the surgery alone and CRT+ surgery groups, respectively (p = 1.0). Median overall survival was 11.1 and 31.4 months in the surgery alone and CRT+ surgery groups, respectively (log rank test, p = 0.042). In the surgery alone group one, 3 and 5 year survival rates were 44%, 24% and 16%, respectively and in the CRT+ surgery group they were 68%, 44% and 29%, respectively. By multivariate analysis we found that age of patient, performance status, alcoholism and > = 4 pathological positive lymph nodes in resected specimen were significantly associated with overall survival, whereas high-dose preoperative CRT was not.

**Conclusion:**

We found no significant survival advantage in esophageal cancer stage IIA-III following preoperative high-dose CRT compared to surgery alone. Patient's age, performance status, alcohol abuse and number of positive lymph nodes were prognostic factors for overall survival.

## Introduction

Patients with esophageal cancer continue to have a poor prognosis with a 5 year survival rate less than 20%. Several factors contribute to this poor outcome, of which the most important is that the vast majority of patients demonstrate either locally advanced or metastatic disease at the time of diagnosis. Surgery has been relatively unsuccessful in controlling loco-regionally-advanced tumors and preoperative concomitant chemotherapy with radiotherapy (RT) followed by resection has become a treatment option. Several studies [1-3] have shown that the prognosis for esophageal cancer patients undergoing surgery might be improved due to the effect of preoperative concomitant chemoradiotherapy (CRT), whereas others have not found any survival benefit by preoperative CRT over surgery alone [4-8]. However, local recurrence and distant metastases remain an issue both after surgery alone and after CRT followed by surgery. In an attempt to improve survival rates, high-dose preoperative CRT was implemented in our hospital from 1996. The applied chemotherapy regimen was originally introduced for the treatment of advanced squamous cell carcinoma of the head and neck, the so-called "Wayne State Regimen" [9]. Improved complete response and survival rates were reported with this regimen which applied cisplatin 100 mg/m^2 ^day 1 and 5-Fluorouracil 1000 mg/m^2^/day, day 1-5 as continuous infusion. Some studies have also suggested a possible positive effect on local tumor control by increasing the RT dose [10-12]. We therefore applied high-dose RT concomitant with intensive chemotherapy (Wayne State Regimen) in an attempt to improve outcome.

The purpose of this study was to investigate the effect of dose intensification of preoperative CRT on overall survival compared to the outcome of surgery alone and possibly also to identify prognostic factors that might influence overall survival.

## Patients and Methods

Two-hundred and one esophageal cancer patients were entered into the database at Haukeland University Hospital, Bergen, Norway during the period 1996 to 2007. In this study we excluded 94 patients due to disease stage 0, I and IV (n = 54), only RT ± surgery (n = 17), definitive CRT due to medical contraindication of surgery (n = 17), only chemotherapy preoperatively (n = 2), different histology than carcinomas (n = 2), sequential chemotherapy and RT preoperatively (n = 1), and gastric cancer during autopsy (n = 1).

The remaining 107 patients were treated with surgery alone (45) or preoperative concomitant high-dose CRT (62). The patients were assigned to surgery alone or CRT followed by surgery according to physician and patient preferences, mainly because survival benefits from preoperative CRT in this study period was considered controversial. Forty-six of 62 patients receiving CRT were deemed resectable before starting CRT and 16 of 62 with T4 tumors deemed resectable pending response to CRT and shrinkage.

Staging of the tumors was performed according to UICC classification (2002) [13] by endoscopic ultrasonography (EUS) and computed tomography (CT) scans of the chest and abdomen. Bronchoscopy was performed in proximally located tumors. Physiological assessment included routine hematological and biochemical assays. Adequate renal and liver functions were required before treatment.

The CRT protocol included three intensive chemotherapy courses concurrent with high-dose RT (66 Gy). Each chemotherapy course consisted of cisplatin 100 mg/m^2^, intravenous infusion over four hours on day 1, and 5-Fluorouracil 1000 mg/m^2^/day as intravenous continuous infusion, on day 1 through day 5. The chemotherapy course was repeated on day 22 and 43. RT was given concomitantly with the second and third chemotherapy courses and was applied as 2 Gy per fraction, 5 fractions per week, 33 fractions in 6.5 weeks to a total dose of 66 Gy. RT was given as CT-based conformal 4 fields' treatment in two phases. Phase 1 RT was given with two anterior-posterior parallel-opposed fields and two lateral oblique fields giving 50 Gy, taking into account the normal tissue tolerance of the spinal cord, heart and lungs. The additional 16 Gy were given using the same four fields, but with different angles for the lateral oblique fields. The gross tumor volume (GTV) was drawn directly onto the axial planning CT images using outlines of the defined primary tumor and nodal disease obtained from the EUS and CT scans. The delineated GTV was the macroscopic tumor including possible macroscopic pathological lymph nodes. The cranial and caudal margins were 3 cm from the GTV and the radial margin was 1.5 cm in the first phase of treatment (50 Gy). After treatment with 50 Gy the radial, cranial and caudal margins were reduced to 1 cm and additional 16 Gy to a total dose of 66 Gy were given. There was no time interval between the two RT phases. About one month following the completion of CRT, a chest and abdomen CT scan and EUS were performed to evaluate treatment outcome.

Toxicities were evaluated and graded according to the National Cancer Institute (NCI) Common Terminology Criteria, version 3.0 [14].

The patients were operated with a right-sided transthoracic or a transhiatal total esophagectomy. All patients had a two-field lymph node resection and left-sided cervical anastomosis, hand-sewn or stapled as of the decision of the surgeon. Most patients had a feeding catheter jejunostomy and feeding was started the day after the operation and continued for 7 - 12 days. All patients had a clinical follow up and underwent radiological and/or endoscopic surveillance when indicated.

### Statistical Analysis

Statistical comparisons between the surgery alone and CRT groups were done with exact chi-square tests and independent samples *t *tests for nominal and continuous variables, respectively. Exact Mann-Whitney U test was used for comparing ordinal as well as unevenly distributed continuous variables. Univariate assessments of categorical prognostic factors for survival and survival analysis were performed using the Kaplan-Meier method with log-rank tests, while continuous risk factors for survival were analyzed by Cox regression survival analysis.

Variables tested for possible influence on survival in univariate analysis were age, gender, smoking, alcoholism, heart disease, lung disease, diabetes mellitus, performance status, hemoglobin level, histology, histological differentiation, tumor (T)-stage at diagnosis, lymph node (N)-stage at diagnosis, disease stage at diagnosis, tumor length, tumor location in esophagus, preoperative CRT, operation method (transthoracic versus transhiatal resection), number of lymph nodes with metastases in resected specimen (no lymph node metastasis, 1-3 nodes with metastasis or 4 or more nodes with metastasis).

Factors found to be significant at univariate analyses were included in multivariate Cox regression survival analysis.

The survival time was calculated from start of treatment (CRT or surgery) to the date of death or to censoring in May 1st 2009.

All p-values are from 2-sided tests, p value ≤ 0.05 was considered statistically significant. All statistical analyses were performed by SPSS 15.0 (SPSS Inc., Chicago, IL, USA). The study was approved by The Regional Committee for Research Ethics in Western Norway.

## Results

Of the 107 patients included in the study there were 94 men and 13 women (median age 65 years, range 39-83). Thirty-nine had squamous cell carcinomas, 67 adenocarcinomas and one had undifferentiated carcinoma. General pretreatment characteristics are shown in Table 1.

**Table 1:** Pretreatment characteristics in 107 esophageal cancer patients.

	Surgery alone n = 45	^#^CRT ± surgery n = 62	p	Surgery alone n = 45	CRT+ surgery n = 31	p
Male/Female	41/4	53/9	0.55	41/4	27/4	0.71
Age median (range), yr	69 (39-83)	63 (46-79)	0.22	69 (39-83)	58 (46-79)	0.032
^† ^PS WHO 0	9 (20)	16 (25.8)	0.60	9 (20)	10 (32.3)	0.08
PS WHO 1	31 (68.9)	42 (67.7)		31 (68.9)	21 (67.7)	
PS WHO 2	5 (11.1)	4 (6.5)		5 (11.1)	0	
Tumor length median (range) cm	5 (1-11.5)	6 (1-12)	0.033	5 (1-11.5)	6 (3-12)	0.035
Upper thoracic tumor	1 (2.2)	10 (16.1)	<0.001	1 (2.2)	1 (3.2)	0.68
Middle thoracic tumor	6 (13.3)	24 (38.7)		6 (13.3)	7 (22.6)	
Lower thoracic tumor	38 (84.4)	28 (45.2)		38 (84.4)	23 (74.2)	
^¶ ^SCC	7 (15.9)	32 (51.6)	<0.001	7 (15.9)	10 (32.3)	0.16
Adenocarcinoma	37 (84.1)	30 (48.4)		37 (84.1)	21 (67.7)	
Disease stage IIA	19 (42.2)	14 (22.6)	0.028	19 (42.2)	8 (25.8)	0.24
Disease stage IIB	6 (13.3)	8 (12.9)		6 (13.3)	6 (19.4)	
Disease stage III	20 (44.4)	40 (64.5)		20 (44.4)	17 (54.8)	
Clinical ^‡ ^T1	0 (0)	2 (3.2)	0.072	0 (0)	1 (3.2)	0.84
Clinical T2	15 (33.3)	13 (21.0)		15 (33.3)	10 (32.3)	
Clinical T3	26 (57.8)	31 (50.0)		26 (57.8)	17 (54.8)	
Clinical T4	4 (8.9)	16 (25.8)		4 (8.9)	3 (9.7)	
Clinical ^§ ^N1	24 (53.3)	42 (73.7)	0.039	24 (53.3)	22 (71)	0.031

^# ^Chemoradiotherapy. ^† ^Performance status. ^¶ ^Squamous cell carcinoma ^‡^Tumor stage, ^§^lymph node stage. One patient in surgery alone group had undifferentiated carcinoma. Percentages in parentheses if not other stated.

There were more smokers in the CRT group than in the surgery alone group (55% versus 34%, p = 0.048) whereas comorbidities (heart disease, lung disease, and diabetes mellitus) and alcoholism were similar in both groups.

Forty-nine patients (79%) received the planned 66 Gy, nine patients (14.5%) received from 60-64 Gy, whereas four patients (6.4%) received between 47.5 and 56 Gy. The mean and median delivered dose-intensities of cisplatin were 84% and 90% of the planned dose, respectively, while mean and median doses of 5-fluorouracil were 86% and 90%, respectively. All chemotherapy dose reductions were due to toxicity.

Median time from end of preoperative CRT to surgical resection was 9 weeks (range 4 to 23 weeks) for patients who were operated on.

### CRT toxicity

CRT toxicity grade 3 occurred in 30 of 62 patients (48.4%) and grade 4 in 24 of 62 patients (38.7%). Toxicity grade 5 (death) occurred in one patient. This patient had grade 5 leucopenia, grade 5 neutropenia, grade 5 thrombocytopenia and died of neutropenic infection after completed CRT.

The following CRT toxicities occurred as both grade 3 and 4: Leucopenia (37 patients), neutropenia (34 patients), neutropenic infection (13 patients), thrombocytopenia (20 patients) and reduced performance status (20 patients).

CRT toxicities that occurred as grade 3 only were esophagitis (35 patients), stomatitis (12 patients), anorexia (23 patients), nausea (22 patients), vomiting (5 patients) and anemia in one patient. Each patient might have several types of toxicity.

### Resectability

Fifty percent (31 patients) in the CRT group did not undergo the planned surgery. The reason for this was still T4 tumor after response to CRT (8), reduced performance status after CRT (8), cerebrovascular accident during the CRT (1), esophageal fistula and technical difficulties (1) and progression of disease with inoperability (13).

In the surgery alone group 25 patients (55.6%) were operated by transthoracic esophagectomy (TTE) and 20 (44.4%) by transhiatal esophagectomy (THE), whereas in the CRT group 29 patients (46.8%) were operated by TTE, 31 (50%) were not operated, one underwent a by-pass operation due to fistula and one underwent abdominal exploration only due to peritoneal carcinomatosis.

In the surgery alone group, 38 of 45 patients (84.4%) had a curative resection defined as no macroscopic and microscopic residual tumors and negative resection margins (R0), six patients (13.3%) had microscopic positive margin in the resected specimen (R1) and one (2.2%) had macroscopic residual disease (R2 resection) with infiltration in the trachea.

Among the 31 operated patients in the CRT group 26 (84% of 31 patients) had R0 resections, three (10%) had R1 resections and two patients (6.4%) had R2 resections.

### Response to CRT

**Figure 1:**
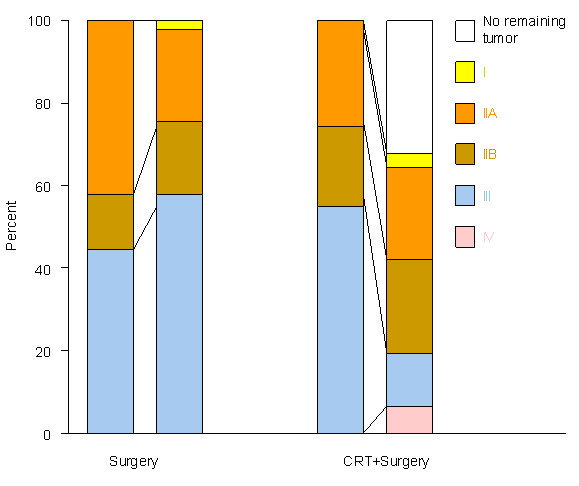
**Stage of disease at diagnosis and after operation; 45 surgery alone and 31 chemoradiotherapy + surgery patients**. CRT = chemoradiotherapy.

Comparison of stage of disease before and after treatment in 31 operated CRT patients demonstrated down-staging in 58%, no change in 23% and up-staging in 19% of the patients (Figure 1 and Table 2).

**Table 2:** Final histopathological stages of tumors and lymph nodes in resected specimens according to treatment group.

	Surgery alone (n = 45)	Chemoradiotherapy+ Surgery (n = 31)^#^	p value
Pathologic ^† ^T0 (%)	0	13 ^‡ ^(41.9)	< 0.001
Pathologic T1 (%)	1 (2.2)	2 (6.5)	
Pathologic T2 (%)	13 (29.0)	8 (25.8)	
Pathologic T3 (%)	29 (64.4)	7 (22.6)	
Pathologic T4 (%)	2 (4.4)	0	
Pathologic ^§ ^N0 (%)	13 (29)	18 (58)	0.027
Pathologic N1, 1-3 positive nodes (%)	19 (42)	9 (29)	
Pathologic N1, ≥ 4 positive nodes (%)	13 (29)	3 (10)	
Pathologic ^¶ ^M1 (%)	0	2 (6.4)	

^# ^One patient in the chemoradiotherapy group who had peritoneal carcinomatosis during operation did not undergo esophagectomy (= TXNX). ^† ^Tumor stage. ^‡ ^Three of these 13 patients had lymph node metastasis. ^§ ^Lymph node stage.^¶ ^Distant metastasis.

Histopathological evaluation of the 31 operated CRT patients demonstrated that 10 patients (32.2%) had pathological complete response (pCR) and 3 patients demonstrated a T0 tumor with 1 or 2 lymph nodes with residual metastasis.

Patients having CRT and not undergoing surgery (31) could only be evaluated clinically. In these patients we found complete response in two (6.4% of 31), partial response in 14 (45.2%) and stable disease in one (3.2%). Progression of the disease was seen in 13 patients (42%) whereof nine had distant metastasis. One patient was not evaluated by CT/EUS after CRT, but had no clinical signs of progressive disease.

In the surgery alone group there was a perfect concordance between preoperative clinical staging and the postoperative pathological staging (Figure 1).

### Postoperative mortality and morbidity

Postoperative mortality and morbidities occurred during 30 days after operation or during the same hospital stay are listed in Table 3. We found no significant differences in morbidity and mortality between the two treatment groups.

**Table 3:** Postoperative mortality and morbidities in 76 esophageal cancer patients.

Complication	Surgery alone, n = 45	^‡^CRT+ surgery, n = 31	P value
Postoperative complications	33	25	0.59
Respiratory failure	20	14	1.0
Pneumonia	19	14	0.82
Anastomosis leakage	5	2	0.69
Wound infection	5	4	1.0
Recurrent laryngeal nerve paralysis	6	5	0.75
Thromboembolism	2	1	1.0
Tracheal injury	0	1	0.41
Bleeding	2	1	1.0
Intraabdominal abscess	1	0	1.0
Chylothorax	0	1	0.41
Other complications	13	9	1.0
Postoperative mortality	4 (9%)	3 (10%)	1.0

^‡ ^Chemoradiotherapy.

### Survival

At time of analysis, 89 of 107 patients had died; follow up time was median 13.6 months for all 107 patients. Dead patients were followed up until death and the alive patients had a median follow-up of 95 months (range 21 - 137 months).

Survival rates for surgery alone (n = 45) and CRT followed by surgery (n = 31) are listed in Table 4. Median overall survival was 11.1 and 31.4 months in the surgery alone and the CRT+ surgery groups, respectively.

**Table 4:** Survival data in 76 esophageal cancer patients (time in months).

	Surgery	95% CI ^†^	^‡ ^CRT + Surgery	95% CI	p
No. of patients	45		31		
Overall survival (median)	11.1	7.63 - 14.53	31.4	11.90-50.98	0.042
Disease-specific survival (median)	11.1	7.63 - 14.53	34.4	6.88-61.91	0.019
Progression free survival (median)	9.5	7.36 - 11.63	18.0	8.48 - 27.52	0.064
DMFS ^¶ ^(median)	9.6	6.75 - 12.50	17.94	6.39 - 29.48	0.058
^# ^LR free survival (median)	X		X		0.67
Patients free of LR after 3 years	69%		71%		
Patients free of LR after 5 years	61%		71%		
1 year overall survival rate	44%		68%		
3 year overall survival rate	24%		44%		
5 year overall survival rate	16%		29%		

^† ^Confidence interval. ^‡ ^Chemoradiotherapy. ^¶ ^Distant metastasis free survival. ^# ^Local recurrence. X = not reached. Local recurrence free survival stands for survival from treatment start until disease recurrence in the field of radiotherapy and/or field of surgery in the mediastinum.

**Figure 2:**
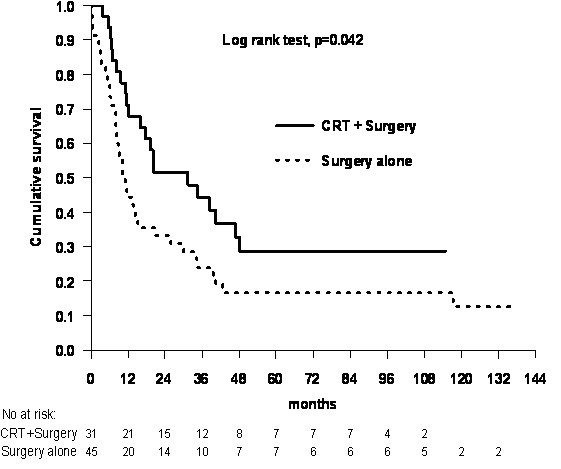
**Overall survival in esophageal cancer following surgery alone or chemoradiotherapy + surgery**. CRT = chemoradiotherapy. Univariate analysis.

**Figure 3:**
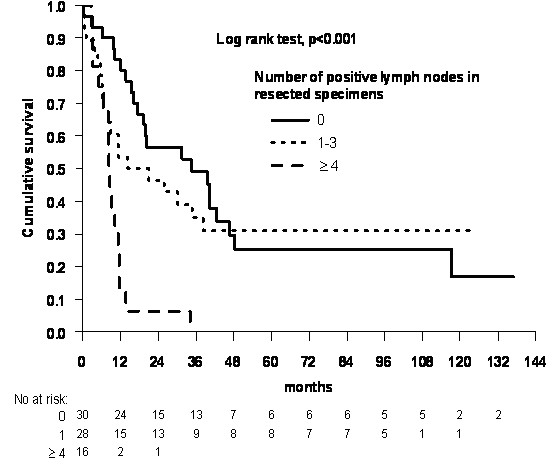
**Survival versus number of lymph node metastasis in resected specimens in 76 esophageal cancer patients**.

By univariate analysis we found that a favorable overall survival was associated with preoperative CRT (p = 0.042, Figure 2), younger age (p = 0.017), better performance status (p < 0.001), no alcoholism (p = 0.028) and TTE (p = 0.048). In addition, ≥ 4 pathologically positive lymph nodes in resected specimens were a negative prognostic factor for survival (p < 0.001, Figure 3). We found no effect on survival of age, gender, smoking, alcoholism, heart disease, lung disease, diabetes mellitus, performance status, hemoglobin level, histology, histological differentiation, T-stage at diagnosis, N-stage at diagnosis, disease stage at diagnosis, tumor length and tumor location in esophagus.

Multivariate analysis showed, however, that age, performance status, alcohol abuse and number of lymph nodes with metastases in operation specimen were significantly associated with overall survival (Table 5).

**Table 5:** Prognostic factors for overall survival in 76 esophageal cancer patients (multivariate Cox regression analysis).

Factors		^† ^HR	95% CI ^‡^		p value
Age		1.04	1.00	1.07	0.029
Performance status	WHO grad 0	1			0.001
	WHO grad 1	1.71	0.83	3.51	0.14
	WHO grad 2	11.96	3.40	42.12	<0.001
Alcoholism	No	1			
	Yes	2.37	1.16	4.84	0.018
Type of esophagectomy	Transthoracic	1			
	Transhiatal	1.20	0.57	2.55	0.63
Positive lymph nodes ^§^	0	1			0.002
	1-3	1.43	0.73	2.81	0.29
	≥ 4	3.84	1.79	8.23	0.001
Treatment	Surgery alone	1			
	Chemoradiotherapy + Surgery	1.02	0.50	2.07	0.95

^† ^Hazard ratio. ^‡ ^Confidence interval. ^§ ^Number of lymph nodes with metastasis in the resected specimen.

Comparing the incidence and type of disease recurrence in the two treatment groups showed a higher rate of distant metastases in surgery alone group (Table 6).

**Table 6:** Site of first recurrence in 76 esophageal cancer patients.

	Surgery alone, n = 45	Chemoradiotherapy + surgery, n = 31
^† ^Locoregional failure	0	2 (6)
Distant metastasis	24 (53)	9 (29)
Local and distant simultaneously	3 (7)	3 (10)
No recurrence	11 (24)	12 (39)
R1 or R2 resections ^‡^	7 (16)	5 (16)

^† ^Locoregional failure stands for disease recurrence in the field of radiotherapy and/or field of surgery in the mediastinum. ^‡ ^Remaining microscopic or macroscopic disease after surgery. Percentages are in parentheses.

## Discussion

In this study, the high-dose preoperative CRT did not demonstrate a significant survival benefit compared to surgery alone by multivariate analysis, although CRT+ surgery patients had longer survival. Several randomized studies have also failed to show a survival advantage following neoadjuvant CRT [4,5,7] and our results are consistent with them although we applied high-dose CRT.

Furthermore, we found that age, performance status, alcoholism and number of positive lymph nodes were significantly associated with overall survival.

However, the preoperative CRT in this study induced response and down-staging of primary tumors, lymph node metastases and combined TNM stages in both operated and non-operated patients (Figure 1, Table 2). This is consistent with report of Kesler et al [15] who have shown that preoperative CRT causes down-staging in esophageal cancer.

In addition, we found that lymph node status is a predictor of outcome where patients with ≥ 4 positive lymph nodes have poorest survival (Figure 3). This is confirmed by previous studies [15-18].

The preoperative CRT was strongly correlated with lymph node stage in resected specimens; this simultaneously with limited number of patients in this study might be factors that contributed to a non-significant p-value of preoperative high-dose CRT when both CRT and number of positive lymph nodes were included in the multivariate Cox regression analysis.

Further, we found no difference in local tumor control between the two treatment groups, and the median local recurrence free survival (survival from treatment start until disease recurrence in the field of radiotherapy and/or field of surgery in the mediastinum) was not reached in both groups (Table 4). The role of CRT and surgery in achieving local tumor control has been disputed [19,20]. However, it should be noted that patients in the CRT group had more lymph node metastases, more advanced stage of disease and longer tumors at diagnosis compared to the surgery alone group. According to this we might expect that preoperative CRT had contributed to an improved local tumor control. At the same time our findings of residual tumors in resected specimens in a large proportion of patients having R0-resections after CRT indicate that esophagectomy is advisable after CRT if R0 resection is possible. Hence, we conclude that both preoperative CRT and radical surgery with extensive lymph node dissection are essential to obtain a good local tumor control.

Our study has, however, some limitations and the results should be interpreted with caution. The study was retrospective with limited number of patients and the treatment groups included both adenocarcinomas and squamous cell carcinomas. This is because at the time of this study, from 1996, the treatment of both histologies was almost the same and only last years the experts are trying to treat them differently.

Another finding in our study was that the high-dose preoperative CRT group had a much higher frequency of serious toxicities compared to other studies applying lower doses of concomitant cisplatin, 5-fluorouracil and RT [21-25]. This should be taken into consideration in further neoadjuvant regimens for esophageal cancer patients.

The main reason for the inferior survival in esophageal cancer patients generally is the early and frequent occurrence of distant metastases. This was also found in our study, as we found a high proportion of distant metastasis in both treatment groups, and highest in the surgery alone group. It is obvious that refinements of chemotherapy or new and more effective systemic treatments, which are able to treat subclinical metastases, are required for these patients.

The observed survival rate in the 31 CRT+ surgery patients in our series was not superior to what is reported in published series which applied lower doses of preoperative CRT [2,5,6,8,15,19,22,26-30]. Due to different patient populations in reported series comparisons between various treatment regimens should be interpreted with caution. However, based on our study and other reported series with pretreatment factors comparable to ours using lower doses of cisplatin and 5-fluorouracil concomitant with RT [2,5,8,15,19,27-30] it is evident that higher doses of CRT is not superior to conventional doses and also have increased toxicities. In consistence with the RTOG 94-05 trial [23] which was published in 2002 we do not recommend high-dose preoperative CRT outside clinical trails.

## Conclusion

Our high-dose preoperative CRT did not show a significant survival advantage over surgery alone and over what is reported in previous studies applying cisplatin, 5-Fluorouracil and RT in conventional doses. Development of new cytotoxic regimens or other systemic therapies are required in order to cure subclinical distant metastases and significantly improve the prognosis. Age, performance status, alcohol abuse and number of positive lymph nodes are significantly associated with overall survival.

## Competing interests

The authors declare that they have no competing interests.

## Authors' contributions

ORM, MH, and AV assisted in the conception and design of the study. MH assisted in the collection and assembly of the data. MH, TWL, AV and KØ assisted in data analysis and interpretation. MH, AV, KØ, TWL, ORM and RS assisted in writing the manuscript. All authors read and approved the final manuscript.
